# 

**Published:** 1888-06

**Authors:** 


### TABLE OF CONTENTS.

### ORIGINAL AND SELECTED ARTICLES.

Laparotomy, with attachment of illum to incision, in a case of volvulus, by J. McFadden Gaston, M.D., Atlanta, Ga., Professor of Surgery, Southern Medical College ..... 201
The age for revaccination ..... 204
Mind Cure, by S. P. Crawford, A. M., M. D., Stockton, California ..... 205
Report of four cases of enterotomy, by Edward von D[illegible]nhoff, M. S., M.D., Athens, Ga ..... 209
The American Medical Association, reported by P. R. Cortelyou, M.D ..... 213
Little things in medicine, by Dr. N. B. Herring, Wilson, N. C ..... 215
Alum in typhoid fever; how to give iron ..... 218

### ABSTRACTS AND GLEANINGS.

A very valuable lesson for those who use anæsthetics ..... 219
Cholecystotomy with recovery ..... 221
The treatment of sleeplessness ..... 222
Antipyrine and Antifebrin ..... 223
Antipyrine for Headaches ..... 224
How to Tampon ..... 225
Good daily diet; tribrimphenol ..... 226
Sulphate of codeine ..... 227
Antifebrin as a nervine; a new method of local refrigeration by chloride of methyl ..... 228
Typhoid in children; antipyrine in labor ..... 229
Lactic acid in the diarrhœas of children; a new and reliable remedy for coccygodynia and pruritus ani ..... 230

### SCIENTIFIC ITEMS.

The time in which we think; The origin of petroleum ..... 231
A new flameless explosive; origin of meteorites ..... 232

### PRACTICAL NOTES AND FORMULÆ.

Cascara sagrada; dysentery; dysentery; pruritus ani; pile ointment ..... 233
Geranium maculatum in chronic bronchitis; Convenient combinations for administering hydrochloric acid in dyspepsia ..... 234

### EDITORIALS AND MISCELLANEOUS.

Editorial brevities ..... 235
Books and pamphlets received ..... 237
Special notes ..... 240

